# Assessing the influence of acute kidney injury on the mortality in patients with acute myocardial infarction: a clinical trail

**DOI:** 10.1080/0886022X.2017.1419969

**Published:** 2018-01-04

**Authors:** Yan-Bei Sun, Yuan Tao, Min Yang

**Affiliations:** a Department of Nephrology, The Affiliated Third Hospital of Soochow University, Changzhou, Jiangsu Province, China;; b Medical Record Room, The Affiliated Third Hospital of Soochow University, Changzhou, Jiangsu Province, China

**Keywords:** Acute kidney injury, mortality, KDIGO, baseline GFR, acute myocardial infarction

## Abstract

**Objectives:** Acute kidney injury (AKI) increases the risk of death following acute myocardial infarction (AMI). In this current study, we tried to understand the role of newly KDIGO defined AKI in AMI-induced early and late mortality.

**Methods:** We retrospectively analyzed the clinical data of AMI patients (totaling 1371 cases) from the hospital’s computer database. And AKI was defined based on the KDIGO criteria but GFR or urinary output assessment was not used. Subsequently, we compared the association of AKI with 30-day and 30-day to 5-year all-cause mortality, using multivariate COX regression analysis with two models.

**Results:** We observed the development of AKI in 410 (29.9%) patients during the hospital stay. The 30-day and 30-day to 5-year mortality rates were 5.6% and 11.3%, respectively, in 1371 AMI patients. Further, adjusted Cox regression analysis based on model 1 revealed that AKI severity was an independent risk factor of 30-day mortality, while AKI Stage 3 was an independent predictor of 30-day to 5-year mortality. Adjusted Cox regression analysis based on model 2 revealed that normal baseline renal function with AKI and impaired renal function with AKI were independent risk factors of 30-day mortality, while normal baseline renal function with AKI and impaired renal function with AKI were identified to be independent predictors of 30-day to 5-year mortality.

**Conclusions:** Whether the baseline renal function decreased or not, AKI strongly correlated with short- and long-term all-cause mortality in patients with AMI. Specifically, the short-term mortality of AMI patients increased with more severe AKI.

## Introduction

Acute kidney injury (AKI) is a common complication observed in hospitalized patients, especially those with acute myocardial infarction (AMI), congestive heart failure, sepsis or undergoing cardiac surgery [1–6]. Recent studies have linked AKI with not only increased in-hospital mortality [1–4,6], but also with short- [4,6,7] and long-term mortality [8–10] after discharge. However, few studies have examined the association between renal dysfunction and clinical outcomes in patients with acute coronary syndrome. In addition, the information about the short- and long-term prognostic value of AKI in Chinese patients with acute myocardial infarction is also not available. Moreover, the previously reported morbidity and mortality of AMI-associated AKI is extremely heterogeneous, and varies with diagnostic criteria for AKI and the studied clinical setting [11].

Recently, three new classification systems have been developed to diagnose AKI. Among these, the Risk, Injury, Failure, Loss, End-Stage Renal Disease (RIFLE) system [12,13] and Acute Kidney Injury Network (AKIN) criteria [14] were the most commonly used systems to study AKI syndrome. Unfortunately, these existing systems, while useful and widely validated, have some limitations. Thus, in 2012, the third and latest classification system was developed by the Kidney Disease: Improving Global Outcomes (KDIGO), an Acute Kidney Injury Work Group [13]. This system included a wider combination of RIFLE and AKIN criteria and defined AKI as an increase in serum creatinine (SCr) levels by ≥0.3 mg/dL within 48 h or an increase of SCr to >1.5 times of the baseline within the prior 7 days; or a urine volume of 0.5 mL/kg/h for 6 h. Rodrigues, Bruetto in 2013, for the first time, stated that KDIGO criteria were more suitable for AKI diagnosis in AMI patients than RIFLE criteria [11]. However, there are still few researches on the role of AMI-associated AKI based on KDIGO classification in prognosis [15,16].

Thus, in this study, we have tried to evaluate the role of AKI as defined by KDIGO during hospitalization, in predicting the early (30-day) and late (30-day to 5-year) mortality of AMI patients.

## Subjects and methods

### Study subjects

This is a single-center observational retrospective cohort study, based on the data from 1655 consecutive patients who were diagnosed with AMI and admitted to the cardiovascular department between December 2008 and December 2012. Patients were included in this study if they were ≥18 years old (two cases were excluded), had at least two SCr measurements in the first 7 days of hospitalization (119 cases were excluded), had minimum Scr value ≥40 μmol/L (19 cases were excluded) and had complete medical record (55 cases were excluded). The patients were excluded if they had more than one hospitalization for AMI (22 cases), had obstructive AKI (three cases), had history of nephrectomy or kidney transplantation therapy for chronic kidney disease (two cases), had eGFR rate of <15 mL/min/1.73 m^2^ on admission or undergone dialysis before admission (31 cases) and had critical illnesses and died within 48 h after admission (31 cases). The final patient cohort consisted of 1371 subjects assessed based on KDIGO criteria.

### Study design

Patients and their primary care physicians made all the clinical decisions. The clinical data collected from the hospital’s computer database included: patient characteristics, past medical history, final discharge diagnosis, electrocardiogram findings, laboratory investigations, echocardiography changes, medical therapies, use of cardiac procedures and interventions, out-hospital outcomes, cardiovascular and overall mortality. The primary endpoint of this study was all-cause mortality, 30-day and 30-day to 5-years after an AMI. The study was approved by the Institutional Ethics Committee of the Third Affiliated Hospital of Soochow University. Informed consent was waived due to the observational nature of our study. The follow-up was performed using Population Death Information Registration and Management System from China Information System for Disease Control and Prevention.

### Definitions

After admission of the patients, their initial estimated glomerular filtration rate (eGFR) was calculated using abbreviated Modification of Diet in Renal Disease (MDRD) study equation [17]. Initial renal dysfunction was defined as a baseline eGFR value of <60 mL/min/1.73 m^2^ >3 months. However, many patients may have AKI without any baseline display of renal function. In these cases, one option was to calculate a theoretical baseline SCr value for a given patient assuming a normal GFR by the simplified MDRD formula. By normalizing the GFR to the body surface area, a GFR of approximately 75–100 mL/min/1.73 m^2^ can be assumed as normal, and any change from this baseline can be estimated for a given patient [12].

The definition of AKI according KDIGO is SCr increase >0.3 mg/dL within 48 h of renal insult or increase >1.5 times baseline within 7 days of admission [13]. GFR and urinary output criteria were not used for AKI diagnosis and staging in this study. AKI stage was defined as following: Stage 1 indicated the increase of SCr levels by 1.5–1.9 times of baseline or ≥0.3 mg/dL increase; Stage 2 represented the increase of SCr levels by 2.0–2.9 times of baseline and Stage 3 depicted 3.0 times increase of SCr levels as compared to baseline or an increase of ≥4.0 mg/dL or initiation of renal replacement therapy. The non-AKI was defined as a change in creatinine level of <0.3 mg/dL.

AMI was diagnosed when there was an observation of two or more following characteristics: chest pain, ischemic ECG change or elevated cardiac marker. This was further classified as ST-elevation MI (STEMI) or non-STEMI (NSTEMI) according to the ECG findings. STEMI was defined as ST-segment elevation by >1 mm in two contiguous leads. Elevated cardiac marker was defined as when there was >3-fold increase in the peak cardiac troponin I levels in comparison to the upper normal limit within 72 h after admission.

### Assessment of cause of death

Cardiovascular (CV) causes of death were sub-classified as myocardial infarction (MI), pump failure, presumed CV death, CV procedural (related to surgical or percutaneous cardiac procedures), other CV causes, etc.

### Statistical analysis

Statistical analyzes were done using the R 2.15.2 software (http://www.r-project.org/, the University of Auckland, New Zealand). The *χ*^2^ test or Fisher’s exact test was used to compare the categorical variables. The ANOVA and the *t*-tests were used for variables with normal distributions, and the Mann–Whitney *U*-test or Kruskal–Wallis test was used for other data. Normally distributed measurement data were expressed as x¯±. Abnormally distributed measurement data were expressed as *M*(1/4, 3/4). Using Kaplan–Meier method, event-free survival was estimated and curves were compared with log-rank test. Predictive factors related to 30-day mortality or 30-day to 5-year mortality were analyzed using univariate and multivariate Cox proportional analyzes. Patients were divided into four groups according to AKI staging in model 1 and admission eGFR and AKI development in model 2, respectively. Analyses were adjusted for age, gender, Killip class ≥2, left ventricular ejection fraction (LVEF), previous medical history (stroke history and hyperuricemia), medicinal therapy (aspirin, beta blocker, angiotensin converting enzyme ACE inhibitors and statin), SBP, DBP and albumin level. The *p* values of less than .05 was deemed as significant.

## Results

### Baseline clinical characteristics of enrolled patients

Table 1 lists the baseline information of all enrolled AMI patients. Among the 1371 (82.8%) effective inpatients, AKI prevalence was 29.9% (410/1371), of which, that was 22.1% (304/1371) for AKI patients at Stage 1, 5.1% (70/1371) for those at Stage 2 and 2.6% (36/1371) for those at Stage 3.

**Table 1.** Baseline clinical characteristics of enrolled patients.

	All patients(*n* = 1371)
Age (years)	67 (57, 76)
Gender	
Male (*n*, %)	1022 (74.5)
Female (*n*, %)	349 (25.5)
Smoking (*n*, %)	496 (36.2)
Cardiovascular disease (*n*, %)	135 (9.8)
Hypertension (*n*, %)	916 (66.8)
Hyperuricemia (*n*, %)	271 (19.8)
Diabetes (*n*, %)	410 (29.9)
Kidney disease (*n*, %)	86 (6.3)
Stroke (*n*, %)	213 (15.5)
Acute infection (*n*, %)	324 (23.6)
Malignant arrhythmias/cardiac syncope/cardiogenic shock attack (*n*, %)	91 (6.6)
STEMI (*n*, %)	863 (62.9)
NSTEMI (*n*, %)	508 (37.1)
Hospital stay (d)	13 (10, 17)
Days in ICU(d)	6 (4, 9)
SBP at admission (mmHg)	133 (118, 150)
DBP at admission (mmHg)	80 (70, 90)
FBG (mmol/L)	6.0 (5.2, 7.5)
Admission eGFR (MDRD) [mL/min/1.73 m^2^]	65.8 (50.8, 78.7)
Hemoglobin (g/L)	131.0 (119.0, 142.4)
Albumin (g/L)	35.3 (32.7, 37.6)
Killip grade (*n*, %)	
Grade I	1110 (81.0)
Grade II	120 (8.8)
Grade III	71 (5.2)
Grade IV	70 (5.1)
LVEF (%)	58 (50, 62)
Aspirin therapy [*n* (%)]	1308 (95.4)
β-Blocker therapy [*n* (%)]	907 (66.2)
ACEI/ARB therapy [*n* (%)]	1094 (79.8)
PCI [*n* (%)]	451 (32.9)
CABG [*n* (%)]	52 (1.9)

Normally distributed measurement data were expressed as x¯±s, abnormally distributed measurement data were expressed as *M*(1/4, 3/4).

SBP: systolic blood pressure; DBP: diastolic blood pressure; FBG: fasting blood glucose; CHD: coronary heart disease; STEMI: ST elevation myocardial infarction; NSTEMI: non-ST elevation myocardial infarction; eGFR: estimated glomerular filtration rate; AKI: acute kidney injury; LVEF: left ventricular ejection fraction; ACEI: angiotensin-converting enzyme inhibitors; ARB: angiotensin II receptor blockers; PCI: percutaneous coronary intervention; CABG: coronary artery bypass graft.

### Comparison of baseline clinical and biochemical characteristics during admission based on KDIGO classification of AKI

We observed no significant differences between the four groups (non-AKI and AKI Stage 1, 2 & 3 groups) of patients for hypertension, smoking, coronary heart disease, ST elevation myocardial infarction (STEMI), non-ST elevation myocardial infarction (NSTEMI) and impaired LVEF. The AMI patients with severe AKI were older women with diabetes mellitus, stroke history, malignant arrhythmias/cardiac syncope/cardiogenic shock attack, hyperuricemia, lower albumin, advanced Killip class, low blood pressure and impaired kidney function at the time of admission, and had not received medicine therapy (including aspirin, β-blocker and ACEI/ARB therapy) and interventional or coronary artery bypass graft therapy

The 5-year all-cause mortality and cardiovascular mortality were significantly higher in patients of AKI Stage 1, 2 & 3 groups than in non-AKI group (all *p* *<* .001). Cardiovascular mortality is the leading cause of death in patients with different AKI staging (Table 2).

**Table 2.** Comparison of baseline clinical and biochemical characteristics during admission based on KDIGO classification of AKI.

	Non-AKI (*n* = 961)	AKI Stage 1 (*n* = 304)	AKI Stage 2 (*n* = 70)	AKI Stage 3 (*n* = 36)	*p* Value
Age (years)	65.0 (56.0, 75.0)	70.0 (59.0, 78.0)*	74.5 (62.0, 80.0)*	69.0 (62.5, 77.3)	<.001
Women [*n* (%)]	199 (20.7)	103 (33.9)*	31 (44.3)*	16 (44.4)*	<.001
Hypertension [*n* (%)]	646 (67.2)	197 (64.8)	47 (67.1)	26 (72.2)	.667
Diabetes [*n* (%)]	252 (26.2)	105 (34.5)*	38 (54.3)*^,^**	15 (41.7)*	<.001
Smoking [*n* (%)]	363 (37.8)	111 (36.5)	14 (20.0)*^,^**	8 (22.2)	.069
CHD [*n* (%)]	79 (8.2)	24 (7.9)	8 (11.4)	1 (2.8)	.511
Stroke history [*n* (%)]	130 (13.5)	60 (19.7)	12 (17.1)	11 (30.6)	.002
Malignant arrhythmias/cardiac syncope/cardiogenic shock attack [*n* (%)]	32 (3.3)	8 (2.6)	10 (14.3)*^,^**	13 (36.1)*^,^**	.001
STEMI [*n* (%)]	602 (62.6)	190 (62.5)	46 (65.7)	25 (69.4)	.768
NSTEMI [*n* (%)]	359 (37.4)	114 (37.5)	24 (34.3)	11 (30.6)	.768
Hyperuricemia [*n* (%)]	142 (14.8)	75 (24.7)*	37 (52.9)*^,^**	17 (47.2)*^,^**	<.001
SBP at admission (mmHg)	135.0 (119.0,151,0)	131.0 (119.8,147.3)*	122.5 (108.5,144.3)*	122.5 (103.8,138.3)	.001
DBP at admission (mmHg)	80 (71,90)	78 (70,87)*	76 (63,85)*	72 (63,83)*	<.001
FBG (mmol/L)	5.61 (5.11,7.27)	6.50 (5.38,8.40)	8.77 (6.40,12.94)	7.61 (6.50,13.71)	<.001
Albumin (g/L)	35.7 (33.2,37.8)	34.5 (32.0,37.0)*	33.1 (29.6,35.9)*	33.2 (29.4,38.1)	<.001
Admission eGFR <60 mL/min/1.73 m^2^ [*n* (%)]	175 (18.2)	78 (25.7)*	37 (52.9)*^,^**	18 (50.0)*^,^**	<.001
LVEF <40% [*n* (%)]	41 (4.3)	9 (3.0)	9 (12.9)*^,^**	3 (8.3)	.300
Aspirin therapy [*n* (%)]	927 (96.5)	289 (95.1)	62 (88.6)*	30 (83.3)	<.001
β-Blocker therapy [*n* (%)]	656 (68.3)	195 (64.1)	38 (54.3)	18 (50.0)	.011
ACEI/ARB therapy [*n* (%)]	779 (81.1)	245 (80.6)*	47 (67.1)	23 (63.9)	.003
PCI therapy [*n* (%)]	346 (36.0)	93 (30.6)	8 (11.4)*^,^**	4 (11.1)*^,^**	<.001
CABG therapy [*n* (%)]	22 (2.3)	18 (5.9)*	6 (8.6)*	6 (16.7)*	.004
Killip class ≥2 [*n* (%)]	156 (16.2)	70 (23.0)	24 (34.2)*	11 (30.5)*	<.001
Mortality					
All-cause mortality [*n* (%)]	114 (11.9)	62 (20.4)*	29 (41.4)*^,^**	18 (50.0)*^,^**	<.001
Cardiovascular mortality [*n* (%)]	62 (6.5)	40 (13.2)*	22 (31.4)*^,^**	17 (47.2)*^,^**	<.001
Cerebrovascular mortality [*n* (%)]	14 (1.5)	8 (2.6)	0 (0.0)	0 (0.0)	N-S
Malignant tumor-related mortality [*n* (%)]	4 (0.4)	2 (0.7)	1 (1.4)	0 (0.0)	N-S
Diabetes complications-related mortality [*n* (%)]	2 (0.2)	2 (0.7)	2 (2.9)	0 (0.0)	N-S
Other cause of mortality [*n* (%)]	17 (1.8)	5 (1.6)	2 (2.9)	1 (2.8)	N-S
Dysoemia [*n* (%)]	15 (1.6)	5 (1.6)	2 (2.9)	0 (0.0)	N-S

Normally distributed measurement data were expressed as x¯±s, abnormally distributed measurement data were expressed as Median (1/4, 3/4 interquartile range).

*p* for no AKI group vs. AKI groups, Chi-square test or Kruskal–Wallis test.

SBP: systolic blood pressure; DBP: diastolic blood pressure; FBG: fasting blood glucose; CHD: coronary heart disease; STEMI: ST elevation myocardial infarction; NSTEMI: non-ST elevation myocardial infarction; eGFR: estimated glomerular filtration rate; AKI: acute kidney injury; LVEF: left ventricular ejection fraction; ACEI: angiotensin-converting enzyme inhibitors; ARB: angiotensin II receptor blockers; PCI: percutaneous coronary intervention; CABG: coronary artery bypass graft.

* *p* < .0083, compared with no AKI group, partitions of chi-square method.

** *p* < .0083, compared with AKI Stage 1 group, partitions of chi-square method.

### Comparison of short- and long-term mortality between non-AKI and AKI patients

AKI patients had a significantly higher 30-day and 30-day to 5-year mortality rates than subjects without AKI (*p* < .05 for all) (Table 3).

**Table 3.** Comparison of short- and long-term mortality between non-AKI and AKI patients.

	Non-AKI	AKI	*p* Value
30-day follow-up (*n* = 1371)	2.2% (21/961)	13.7% (56/410)	<.001
30-day to 5-year follow-up (*n* = 1294)	9.9% (93/940)	15.0% (53/354)	.013

AKI: acute kidney injury.

### Comparison of demographic and clinical characteristics between the non-death group and the death group

The 30-day follow-up revealed no significant differences, between the patients who either survived or died, for hypertension, coronary heart disease, STEMI, NSTEMI, systolic blood pressure and coronary artery bypass graft (CABG) therapy. However, majority of the patients who died, were elderly women with diabetes, had stroke history, showed impaired left ventricular ejection fraction (LVEF <40%), had high fasting blood glucose. In addition, these patients showed impaired baseline eGFR, and AKI along with severe KDIGO classification during hospitalization. Moreover, the death group patients had low diastolic blood pressure, low rates of smoking history and displayed low albumin level, and did not received aspirin therapy, β-blocker therapy, ACEI/ARB therapy or interventional therapy (Table 4).

**Table 4.** Comparison of demographic and clinical characteristics between the non-death group and the death group.

	30-day follow-up			30-day to 5-year follow-up		
	Non-death (*n* = 1294)	Death (*n* = 77)	*p* Value	Non-death (*n* = 1148)	Death (*n* = 146)	*p* Value
Age (years)	66 (28,75)	77 (72,84)	<.001	65 (55,74)	76.5 (69.3,81.0)	<.001
Women [*n* (%)]	313 (24.2)	36 (46.8)	<.001	263 (22.9)	50 (34.2)	.0036
Hypertension [*n* (%)]	859 (66.4)	57 (74.0)	.208	757 (65.9)	102 (69.9)	.394
Diabetes [*n* (%)]	377 (29.1)	33 (42.9)	.015	310 (27.0)	67 (45.9)	<.001
Smoking [*n* (%)]	483 (37.3)	13 (16.9)	<.001	448 (39.0)	35 (24.0)	<.001
CHD [*n* (%)]	101 (7.8)	11 (14.3)	.053	77 (6.7)	24 (16.4)	<.001
Stroke history [*n* (%)]	191 (14.8)	22 (28.6)	.002	150 (13.1)	41 (28.0)	<.001
LVEF <40% [*n* (%)]	56 (5.0)	6 (21.4)	.003	46 (4.6)	10 (8.8)	.082
STEMI [*n* (%)]	821 (63.4)	42 (54.5)	.147	752 (65.5)	69 (47.3)	<.001
NSTEMI [*n* (%)]	473 (36.6)	35 (45.5)	.147	396 (34.5)	77 (52.7)	<.001
SBP at admission (mmHg)	133 (118,150)	130 (117,150)	.458	133 (118,150)	138 (120,153)	.135
DBP at admission (mmHg)	80 (71,90)	71 (65,82)	<.001	80 (71,90)	80 (70,90)	.765
FBG (mmol/L)	5.92 (5.17,7.50)	7.38 (6.08,12.76)	<.001	5.85 (5.14,7.50)	6.50 (5.46,8.00)	.009
Albumin (g/L)	35.5 (32.9,37.7)	32.4 (29.2,35.0)	<.001	35.7 (33.3,37.8)	33.3 (30.4,36.0)	<.001
Admission eGFR <60 mL/min/1.73 m^2^ [*n* (%)]	264 (20.4)	44 (57.1)	<.001	198 (17.2)	66 (45.2)	<.001
AKI [*n* (%)]	354 (27.4)	56 (72.7)	<.001	301 (26.2)	53 (36.3)	<.013
Admission eGFR ≥60 mL/min/1.73 m^2^ without AKI	777 (98.9)	9 (1.1)	<.001	727 (93.6)	50 (6.4)	<.001
Admission eGFR <60 mL/min/1.73 m^2^ without AKI	163 (93.1)	12 (6.9)		120 (73.6)	43 (26.4)	
Admission eGFR ≥60 mL/min/1.73 m^2^ with AKI	253 (91.3)	24 (8.7)		223 (88.1)	30 (11.9)	
Admission eGFR <60 mL/min/1.73 m^2^ with AKI	101 (75.9)	32 (24.1)		78 (77.2)	23 (22.8)	
KDIGO classification [*n* (%)]						
Absence of AKI	940 (72.6)	21 (27.3)	<.001	847 (73.8)	93 (63.7)	.002
AKI Stage 1	276 (21.3)	28 (36.4)		242 (21.1)	34 (23.3)	
AKI Stage 2	53 (4.1)	17 (22.1)		41 (3.6)	12 (8.2)	
AKI Stage 3	25 (2.0)	11 (14.2)		18 (1.5)	15 (4.8)	
Aspirin therapy [*n* (%)]	1252 (96.7)	56 (72.7)	<.001	1115 (97.1)	137 (93.9)	.045
β-Blocker therapy [*n* (%)]	888 (68.6)	19 (24.7)	<.001	801 (69.8)	87 (59.6)	.016
ACEI/ARB therapy [*n* (%)]	1062 (82.1)	32 (41.6)	<.001	948 (82.6)	114 (78.1)	.223
PCI therapy [*n* (%)]	447 (34.5)	4 (5.2)	<.001	437 (38.1)	10 (6.8)	<.001
CABG therapy [*n* (%)]	48 (3.7)	4 (5.2)	.654	44 (3.8)	4 (2.7)	.520

Normally distributed measurement data were expressed as x¯±s, abnormally distributed measurement data were expressed as *M*(1/4, 3/4).

SBP: systolic blood pressure; DBP: diastolic blood pressure; FBG: fasting blood glucose; CHD: coronary heart disease; eGFR: estimated glomerular filtration rate; AKI: acute kidney injury; LVEF: left ventricular ejection fraction; STEMI: ST elevation myocardial infarction; NSTEMI: non-ST elevation myocardial infarction; ACEI: angiotensin-converting enzyme inhibitors; ARB: angiotensin II receptor blockers; PCI: percutaneous coronary intervention; CABG: coronary artery bypass graft.

Similarly, the patients of 30-day to 5-year follow-up group, also showed no significant differences for hypertension, impaired LVEF, systolic and diastolic blood pressure, total cholesterol, ACEI/ARB therapy and CABG therapy, between those who either survived or died. In addition, patients in the death group were more likely elderly women with diabetes, coronary heart disease, stroke history, NSTEMI, high fasting blood glucose, impaired baseline eGFR, AKI and severe KDIGO classification during hospitalization. These patients had also low rates of smoking history, low albumin and did not received aspirin therapy, β-blocker therapy or interventional therapy. Chi-square test also suggested a statistical difference of long-term mortality between patients with and without STEMI (STEMI vs. NSTEMI: 47.3% vs. 52.7%, *p* < .001) (Table 4).

### Multivariate Cox regression analyzes of independent predictors in 30-day and 30-day to 5-year mortality groups

Multivariate Cox regression analysis based on model 1 revealed that, for 30-day mortality, aging, stroke history, malignant arrhythmias/cardiac syncope/cardiogenic shock attack, hyperuricemia and AKI severity were independent risk factors, while albumin level, aspirin therapy, β-blocker therapy and ACEI/ARB therapy were independent protective factors. For 30-day to 5-year mortality, aging, stroke history and AKI Stage 3 were independent risk factors, while albumin level, β-blocker therapy and PCI were independent protective factors (Table 5).

**Table 5.** Multivariate cox regression analysis for 30-day and 30-day to 5-year mortality.

	30-day Mortality			30-day to 5year Mortality		
	HR	95% CI	*p* Value	HR	95% CI	*p* Value
Model 1						
Aging	1.069	1.018–1.123	.007	1.058	1.036–1.080	<.001
Women	1.082	0.456–2.571	.858	0.831	0.542–1.275	.396
LVEF <40%	1.301	0.351–4.823	.694	1.007	0.509–1.994	.984
Stroke history	3.868	1.411–10.597	.009	1.601	1.049–2.445	.029
Malignant arrhythmias/cardiac syncope/cardiogenic shock attack	26.110	4.477–91.187	<.001	1.548	0.533–4.498	.422
STEMI	1.209	0.457–3.202	.702	1.328	0.893–1.975	.162
Hyperuricemia	5.849	2.171–15.761	<.001	1.372	0.856–2.199	.188
Albumin level	0.913	0.834–0.999	.048	0.923	0.877–0.973	.003
FBG at admission	0.983	0.894–1.081	.720	1.003	0.948–1.062	.906
SBP at admission	1.008	0.989–1.026	.410	1.004	0.997–1.011	.278
Admission eGFR <60 mL/min/1.73 m^2^	0.633	0.242–1.657	.352	1.087	0.684–1.726	.724
KDIGO classification						
Absence of AKI	1.000	–	–	1.000	–	–
Stage 1	3.344	1.186–9.433	.023	1.076	0.680 –1.701	.755
Stage 2	6.320	2.206–23.305	<.001	1.705	0.798–3.643	.168
Stage 3	7.922	2.321–35.252	<.001	3.593	1.305–9.892	.013
Aspirin therapy	0.300	0.100–0.897	.031	0.472	0.185–1.203	.116
β-Blocker therapy	0.133	0.047–0.375	<.001	0.644	0.434–0.955	.028
ACEI/ARB therapy	–	–	–	0.855	0.525–1.392	.530
PCI	1.714	0.474–6.203	.412	0.282	0.144–0.555	<.001
CABG	10.010	0.996–42.021	.052	0.773	0.182–3.290	.728
Model 2						
Aging	1.067	1.018–1.118	.007	1.055	1.034–1.078	<.001
Women	1.020	0.436–2.398	.965	0.857	0.560–1.131	.477
LVEF <40%	1.083	0.325–3.611	.897	1.029	0.527–2.011	.933
Stroke history	4.157	1.538–11.238	.005	1.610	1.054–2.460	.028
Malignant arrhythmias/cardiac syncope/cardiogenic shock attack	23.478	7.259–75.937	<.001	1.527	0.523–4.456	.438
STEMI	1.067	0.415–2.744	.893	1.356	0.910–2.021	.135
Hyperuricemia	5.301	2.059–13.647	<.001	1.541	0.980–2.421	.061
Albumin level	0.909	0.828–0.998	.045	0.929	0882 –0.978	.005
FBG at admission	0.974	0.888–1.068	.570	1.015	0.963–1.070	.577
SBP at admission	1.010	0.992–1.028	.268	1.003	0.995–1.010	.495
Admission eGFR ≥60 mL/min/1.73 m^2^ without AKI	1.000	–	–	1.000	–	–
Admission eGFR <60 mL/min/1.73 m^2^ without AKI	0.519	0.102–3.022	.721	1.505	0.910–2.421	.113
Admission eGFR ≥60 mL/min/1.73 m^2^ with AKI	4.352	1.521–15.302	.035	1.682	1.123–2.657	.018
Admission eGFR <60 mL/min/1.73 m^2^ with AKI	6.353	1.699–26.102	.001	1.870	1.144–3.113	.040
Aspirin therapy	0.323	0.113–0.922	.035	0.485	0.191–1.231	.128
β-Blocker therapy	0.137	0.048–0.387	<.001	0.648	0.439–0.958	.030
ACEI/ARB therapy	–	–	–	0.825	0.508–1.340	.435
PCI	1.636	0.455–5.890	.451	0.282	0.143–0.554	<.001
CABG	11.878	0.533–43.569	.058	0.902	0.216 –3.772	.888

SBP: systolic blood pressure; DBP: diastolic blood pressure; FBG: fasting blood glucose; CHD: coronary heart disease; eGFR: estimated glomerular filtration rate; AKI: acute kidney injury; LVEF: left ventricular ejection fraction; STEMI: ST elevation myocardial infarction; NSTEMI: non-ST elevation myocardial infarction; ACEI: angiotensin-converting enzyme inhibitors; ARB: angiotensin II receptor blockers; PCI: percutaneous coronary intervention; CABG: coronary artery bypass graft.

Multivariate Cox regression analysis based on model 2 revealed that, for 30-day mortality, aging, stroke history, malignant arrhythmias/cardiac syncope/cardiogenic shock attack, hyperuricemia, normal baseline renal function with AKI and impaired renal function with AKI were independent risk factors, while albumin level, aspirin therapy, β-blocker therapy and ACEI/ARB therapy were independent protective factors. For 30-day to 5-year mortality, aging, stroke history, normal baseline renal function with AKI and impaired renal function with AKI were independent risk factors, while albumin level, β-blocker therapy and PCI were identified to be independent protective factors (Table 5).

### Assessment of 5-year follow-up prognosis

We noticed that during 1,839,109 person-days of follow-up (mean, 1341.4 ± 600.2 days), 223 patients (16.3%) died.

In model 1, the 5-year incidence of death per 10,000 person-days of follow-up was 0.83 in non-AKI patients, 1.65 in AKI Stage 1, 4.63 in AKI Stage 2 and 6.47 in AKI Stage 3 patients. In model 2, the 5-year incidence of death per 10,000 person-days of follow-up was 0.41 in patients of admission eGFR ≥60 mL/min/1.73 m^2^ without AKI, 1.07 in patients of admission eGFR <60 mL/min/1.73 m^2^ without AKI, 1.72 in patients of admission eGFR ≥60 mL/min/1.73 m^2^ with AKI and 2.27 in patients of admission eGFR <60 mL/min/1.73 m^2^ with AKI.

**Figure 1.**
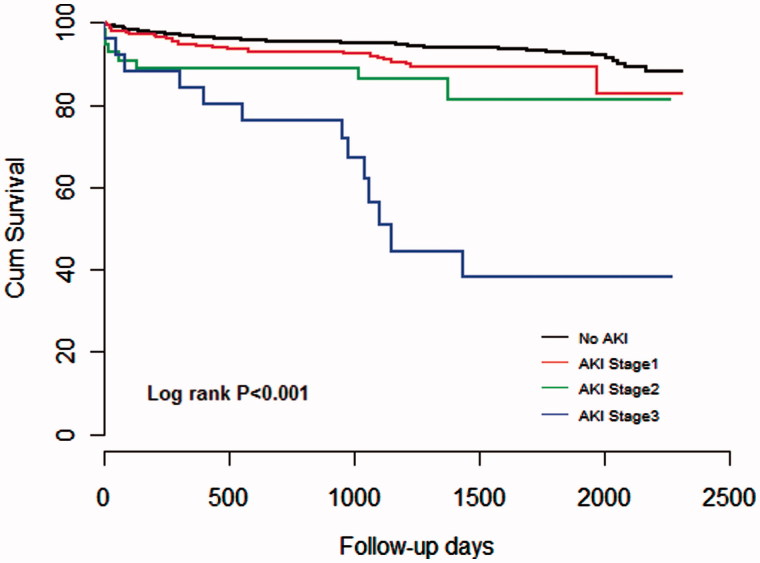
Cox survival curve at 5 years among the four groups according to the different KDIGO defined AKI staging.

**Figure 2.**
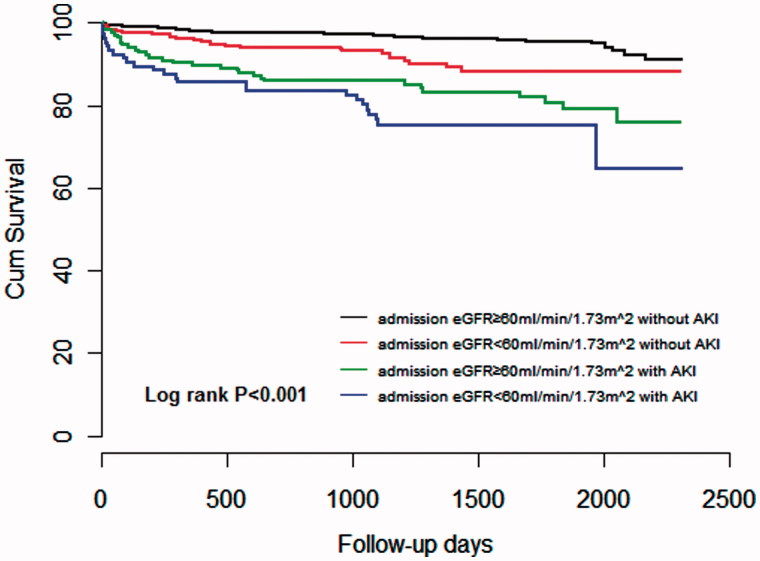
Cox survival curve at 5 years among the four groups divided into admission eGFR and AKI development.

The Kaplan–Meier survival plot and log-rank test showed that all-cause mortality during the 5-year follow-up period was proportional to the severity of AKI determined using KDIGO criteria during hospitalization (Figure 1), and to the admission eGFR and AKI development (Figure 2).

## Discussion

Recent epidemiological studies have shown that the incidence of AMI-associated AKI is increasing at an alarming rate, affecting from 10% to 55% of the patients during hospitalization [15]. Lately Rodrigues, Bruetto et al. stated that KDIGO criteria were more suitable for AKI diagnosis in AMI patients than RIFLE criteria, and they obtained the prevalence of KDIGO-defined AKI was 36.6% [11]. Our study defined AKI and its grading according to KDIGO guidelines and worked out that 410 out of 1371 (29.9%) patients with AMI developed AKI, similar to the above findings.

To best of our knowledge, the follow-up of our patients was longer than that reported in most other studies. Previous studies have suggested that either an impaired renal function at the time patient admission to the hospital or subsequent development of AKI, negatively affects the outcome of patients suffering from an AMI [8,10,18–20]. Bruetto, Rodrigues et al. suggested that early mortality was predominantly associated with the effects of AKI, while long-term outcomes were influenced by AKI development in addition to baseline impaired renal function [15]. In our study, the development of AKI was the predominant factor associated with increased mortality, however, the impaired admission eGFR was negatively associated with an increased mortality in the respective subgroups during both short- and long-term observation. We further provided that the mortality rate was related to the severity of AKI.

Short-term worse outcomes can be explained by the effects of decreased kidney function, such as volume overload and retention of uremic toxins [21]. On the question of how to explain the existing relationship between AKI and long-term mortality, some observational studies have shown that AKI leads to new CKD, regardless of the cause of the AKI, and resulted in an increased long-term risk of end-stage renal (ESRD), and excess mortality [22–24]. The study by Bull et al. has pointed out that renal blood flow and clearance function can remain impaired for a prolonged period of time after an episode of AKI, despite apparent normalization of SCr [25]. It was hypothesized that the development of CKD is one of the potential mechanism of explaining this relationship [26]. Consistent with this, few other studies have also shown an ongoing progressive damage after AKI which resulted in reduced capillary density of peritubular capillaries, a process known as ‘rarefaction’ and linked with the development of CKD, often with a delayed increase in SCr [27].

Therefore, based on our study, we hypothesized that early mortality was largely related to the effects of AKI, while lower long-term survival rates might be influenced to a greater extent by higher stages of AKI-associated ‘new CKD’ than an original renal insufficiency. However, our findings did not justify the causal relationship between AKI and prognosis. Whether AKI is associated with an increase in the prevalence of long-term ESRD and an increase in mortality, remains to be further explored.

It has been widely recognized that follow-up of kidney function is important, but our data highlight the importance of cardiovascular follow-up. In our study, both all-cause mortality and cardiovascular mortality developed more frequently in the AKI group than the control group. Patients who survived an episode of AKI were also at risk for major adverse cardiovascular events, as well as for progression to CKD, regardless of whether there was underlying cardiovascular disease [22,28]. This raised an important question about how to explain this increased risk for cardiovascular events. Currently, we speculate that this might be mediated, especially in the long-term, by development of CKD after AKI, but the remaining impact of developed AKI may directly increase the risk of cardiovascular disease due to involvement of inflammatory or other pathways [25,29,30]. In the acute phase, AKI usually leads to acute cardiac events and has been termed as cardiorenal syndrome type 3 (CRS-3) [31]. At present this concept is only sparsely supported by the human data. However, several studies (follow-up 1–10 years) have shown that AKI survivors are at increased risk for myocardial infarction and heart failure in the years following ICU and non-ICU discharge [32].

Previous study on the Global Registry of Acute Coronary Events (GRACE) project has found that patients with NSTEMI have higher mortality compared with patients with STEMI, presumably due to the more pronounced co-morbidity as well as the more frequent multivessel disease of the former patients [33]. Our results also suggested a statistical difference of long-term mortality between patients with and without STEMI (47.3% vs. 52.7%, *p* < .001). However, multivariate COX regression did not suggest that STEMI was an independent risk factor for mortality. We speculated that it was associated with the lower proportion of NSTEMI distribution. In our study, the STEMI/NSTEMI proportion was obviously higher than that of the GRACE project [863/508 (63%/37%) vs. 3693/2935 (56%/44%)].

In addition, based on multivariate analysis, our study identified that lower albuminemia was significantly associated with higher both short- and long-term mortality. Previous studies have suggested that in patients with severe AKI, plasma albumin levels can also be used as a predictor of mortality, not just a nutritional index [34]. However, albumin, a classic malnutrition marker, has been observed to lose its accuracy in AKI patients, since the reduction in its levels was not always a consequence of the limited energy and protein substrate intake, and thus indicated the presence of inflammation [35,36]. Thus, whether hypoalbuminemia can be an independent predictor of death following AKI development in patients with AMI remains to be fully defined.

PCI treatment was another factor identified in our study, having statistically significant protective effects for the long-term prognosis of patients with AMI-associated AKI. But in general, the contrast medium is nephrotoxic, and may cause acute tubular necrosis [37]. AMI patients with PCI therapy are more likely to suffer from deterioration of renal function than those without, due to the risk of contrast induced nephropathy or contrast-induced AKI (CI-AKI), which make the pathophysiology more complex. Interestingly, among AMI patients across 56 US centers from Cerner Corporation's Health Facts database, the incidence of AKI has progressively declined (from 26.6% in the year 2000 to 19.7% in 2008), as the use of PCI has progressively increased (from 32.1% in the year 2000 to 47% in 2008) [38]. Therefore, CI-AKI seemed not to be the main cause of AKI in patients with AMI. Besides, in this study, PCI was performed only in 33% of all AMI patients, and the treatment rates in severe AKI patients and deaths were significantly lower, which might partly result in that PCI be a protective factor of long-term mortality. The reason why PCI was not an independent protective factor of short-term mortality was supposed to be related to some confounding factors.

Shacham et al. pointed out that patients with AKI had significantly lower systolic ejection fraction (EF; 48% ± 8% vs. 41% ± 10%, *p* < .001), and LVEF emerged as an independent predictor of AKI in multivariate regression analysis [39]. The prevalence of AKI, elevated with the decreased LVEF, can be explained by a dramatic decrease in cardiac output after AMI, resulting in a decrease in renal perfusion, as well as the activation of renin angiotensin–aldosterone system (RAS) and cascade actions. Our prophase research also found lower LVEF levels in AKI patients [55 (48, 61) vs. 58 (51, 62), *p* < .001], but multivariate regression equation did not suggest an independently association with AKI [40]. In this study, LVEF was not even an independent factor for mortality among AMI patients. It is speculated that this may be related to the higher proportion of right ventricular infarction. However, our data did not confirm the data of right ventricular function in echocardiography, which needs further exploration.

A large cohort study revealed that prescription of RAS blockers was associated with only a small increase in AKI risk while individual patient characteristics are much more strongly associated with the rate of AKI [41]. In this study, critically ill AKI patients were less likely to receive ACEI/ARB. Since RAS inhibition post-AMI was associated with lower risk of all-cause mortality, the deficiency of ACEI/ARB in patients with AMI-related AKI may have affected the prognosis.

Our study had number of limitations. First, the primary endpoint of this study was all-cause mortality after an AMI, and we did not collect the information on the progression of kidney disease. Second, because of methodological limitations inherent in the retrospective analyzes, our data could not include the volume of contrast. Thus, we could not address the influence of contrast volume that was administered during percutaneous coronary intervention. Third, this study used the KDIGO criteria to define AKI, but information on the urinary output was not available in all samples. Finally, due to the retrospective cohort design we could not make causal inferences. Residual confounding due to unknown comorbidities or complications or such as multimorbidity during hospitalization or after discharge could have influenced short- and long-term mortality.

In conclusion, our study revealed that AKI occurred in 29.9% of the patients hospitalized for AMI. AKI appeared to be strongly correlated with short- and long-term all-cause mortality, regardless of the baseline renal impairment. In particular, we identified a dose–response relationship between AKI severity and short-term mortality.
